# Supplementary material for: MafB, WDR77, and ß-catenin interact with each other and have similar genome association profiles
Source: PLoS One. 2022 Apr 28;17(4):e0264799. doi: 10.1371/journal.pone.0264799 (PMC9049301; doi:10.1371/journal.pone.0264799)

Figure 1a B-cat WDR77

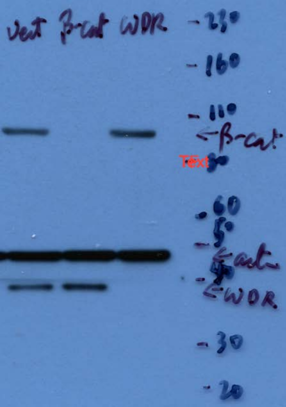

Figure 1a CRISPR MAFB KO

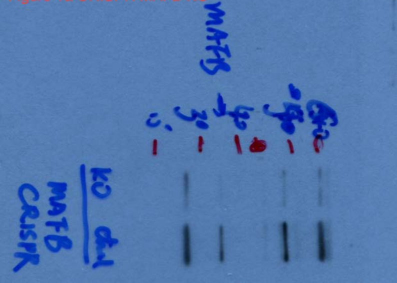

Figure 1a CRISPR MAFB actin

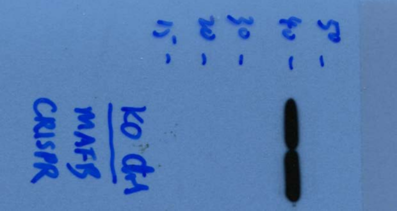

Figure 2A Anti-BCAT IP see b-catenin

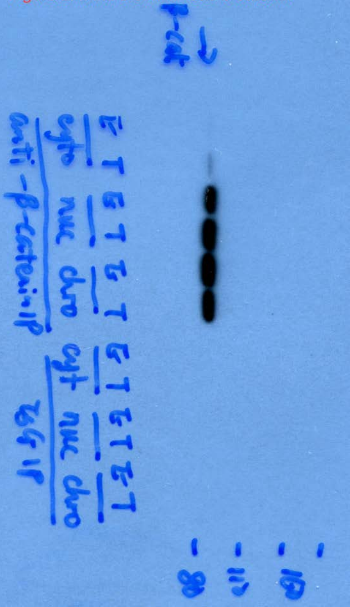

Anti-BCAT IP see MafB

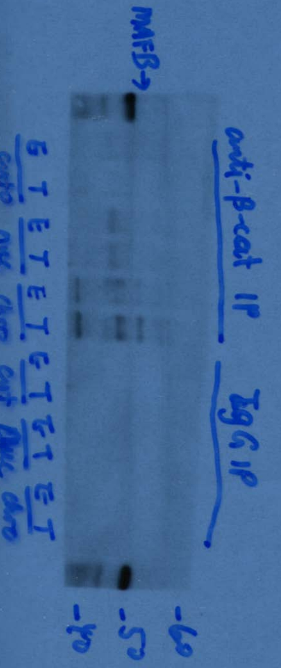

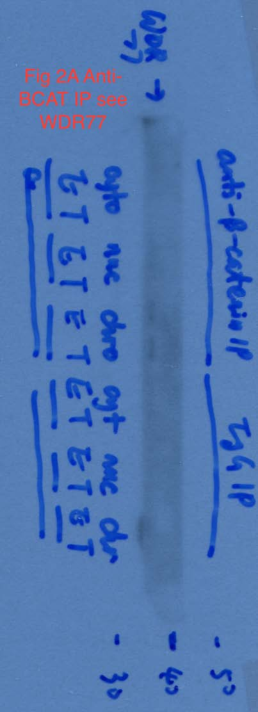

Fig 2A Anti-BCAT IP see WDR77

Fig. 2A- Anti-MafB IP
see BCAT

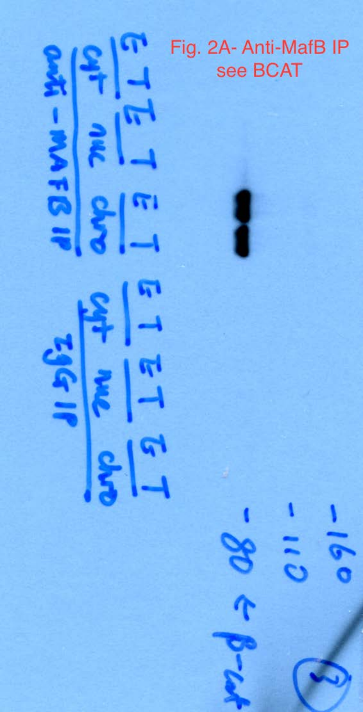

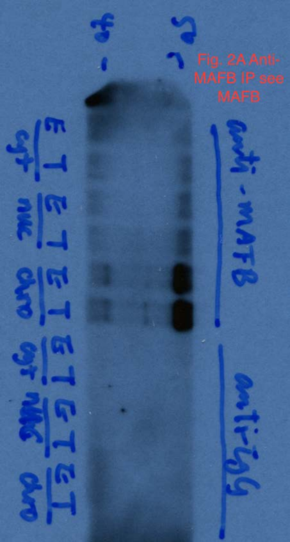

Fig. 2A Anti-MAFB IP see MAFB

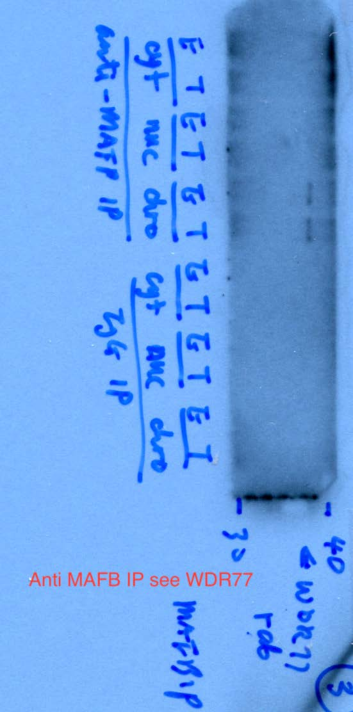

Anti MAFB IP see WDR77

Fig. 2A- Anti-WDR77 IP
see B Catenin

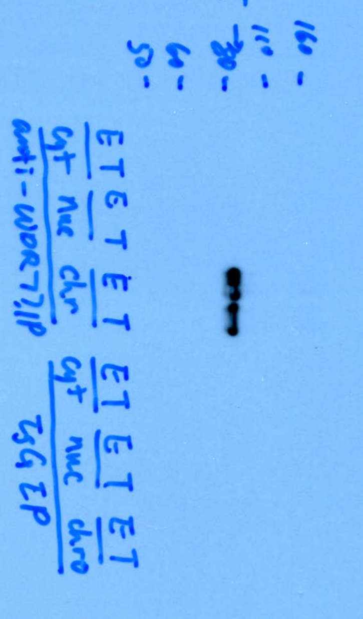

Fig. 2A Anti-WDR77 IP see MAFB

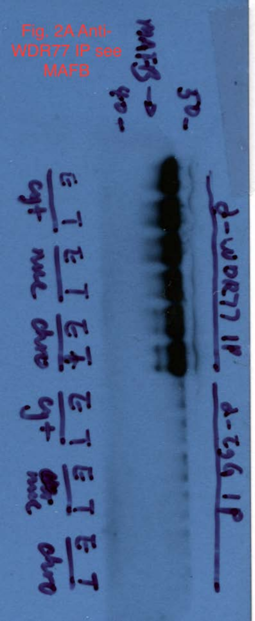

Fig. 2A- Anti-WDR 77 IP
see WDR77

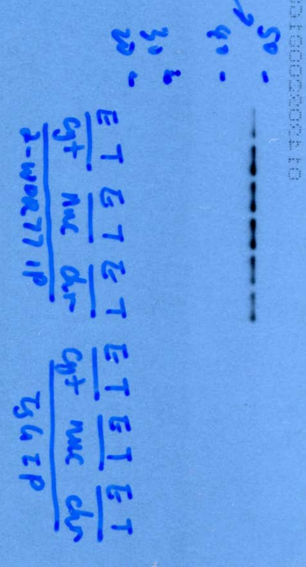

Figure 2B. Lysis B-catenin

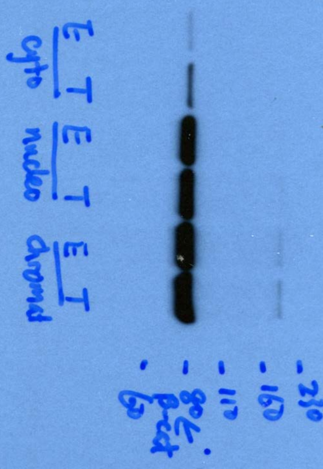

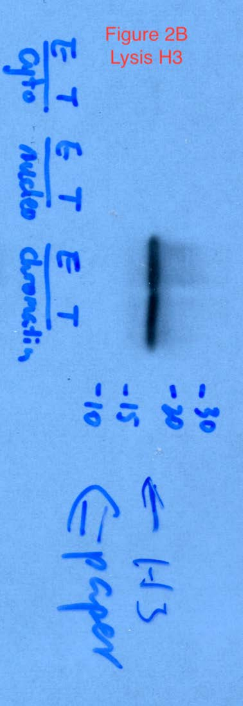

Figure 2B
Lysis H3

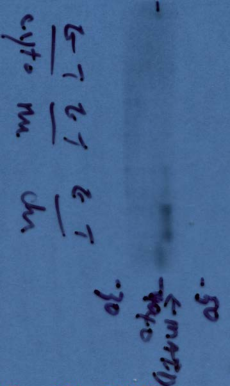

Figure 2B Lysis MAFB

Figure 2B Lysis MEK

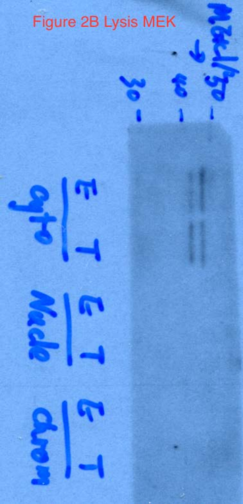

Figure 2B
Lysis U1 SnRNP

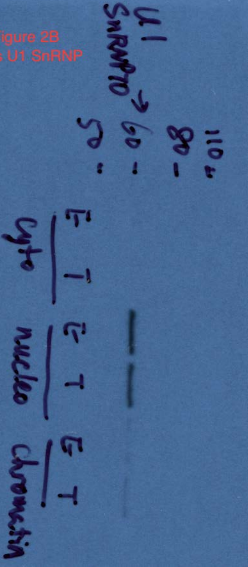

Figure 2B Lysis WDR77

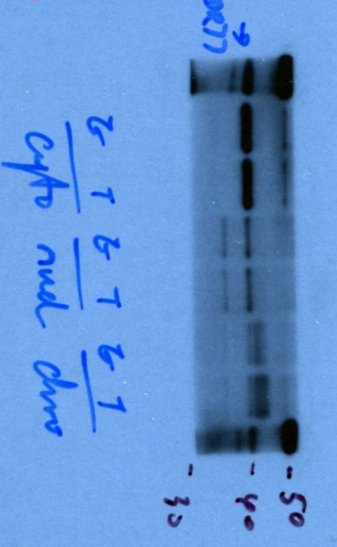

Figure 2C B-catenin-MAFB IP

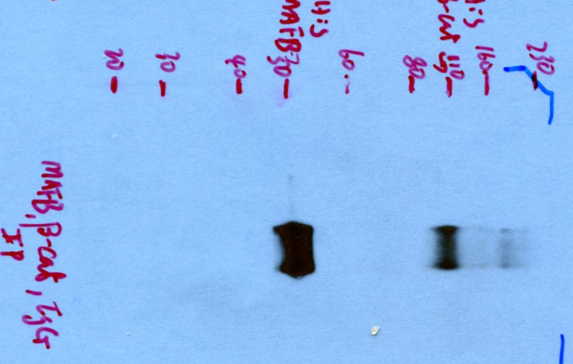

Figure 2C- B-catenin-MAFB lysis

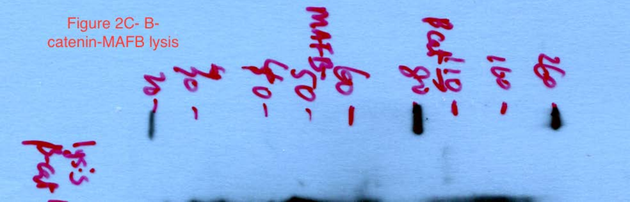

Figure 2C- B-catenin-WDR77 IP

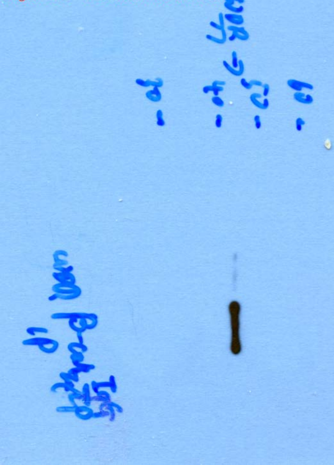

Figure 2C B-catenin-WDR77 lysis

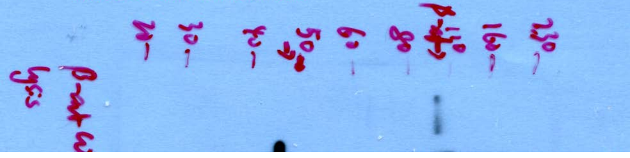

Supplement: S1 Raw images — (PDF) [file pone.0264799.s001.pdf]
