# Supplementary material for: MafB, WDR77, and ß-catenin interact with each other and have similar genome association profiles
Source: PLoS One. 2022 Apr 28;17(4):e0264799. doi: 10.1371/journal.pone.0264799 (PMC9049301; doi:10.1371/journal.pone.0264799)
Supplement: Attachment — Submitted filename: 122668_1_rebuttal_2283878_r6qwn4.doc [file pone.0264799.s002.doc]

February 3, 2022

Dear Editors,

Attached is a revised version of our paper still entitled “**MafB, WDR77, and ß-catenin interact with each other and have similar genome association profiles**” (RC2021-01184) that we are submitting for publication in PLoS One. For reasons discussed below, no new experiments are included, and the revised version is only slightly changed from the original submission. Point-by-point responses below.

**General comments**

The 3 reviews are rather different, but none of them contest the key observations in the title and abstract.

Reviewer 3 clearly understands the strengths and weakness of the paper. The strength of the paper lies with the observations themselves, which are surprising, clearly demonstrated, and potentially interesting. The weakness of the paper is that we don’t understand the functional significance of the results, although we make some suggestions. The paper makes this very clear including the possibility of fortuitous and functionally irrelevant connections. We agree with Reviewer 3 that the paper is worthy of publication, and believe that PLoS is the appropriate journal

Reviewer 1 also thinks that the observations are interesting and presumably worthy of publication so that they can be followed up and/or linked to other observations. This reviewer does not contest any of the observations, all of which involve very standard methodologies. Essentially all the comments are requests to do additional (and often inferior) experiments to make the same observations. We (and Reviewer 3) do not think these are necessary, and indeed believe they would be a waste of time and money. Hence, we did not do them

Reviewer 2 does not contest any of the observations but misunderstands our conclusions. Essentially all the criticisms have to do with the functional significance of the observations, not the observations *per se*. These criticisms are moot, because we never claimed to demonstrate functional significance, and on multiple occasions were clear about the possibility of functional irrelevance. Of course, our lack of proof of functional significance lowers the interest of the paper, and hence the appropriate journal. We (and Reviewers 1 and 3) strongly believe that papers like ours should be published so that the observations can be linked to others and followed up upon. PLoS One is designed to publish such papers, and we believe it to appropriate and acceptable for this paper.

**Reviewer 1**

0. The summary has several errors. First, our previous work had nothing at all about WDR77 and ß-catenin (including the interaction which started the present paper), and there was only one limited experiment on MafB, which was cited. Second, our ER-Src model is hardly questionable. We have published 33 papers using this model, and these papers have been cited nearly 6000 times. Third, we did not use a “sphere formation assay”, which is conventionally done under special growth conditions, but rather a growth in low attachment assay that is essentially the same as the standard soft-agar assay for transformation.

1. Figure 2B shows the level of the 3 proteins in both conditions.

2. Fig. 1B is not a sphere forming assay, but rather a standard transformation assay of growth in low attachment. It is equivalent to the standard soft-agar assay but done slightly differently by a well validated and much easier to do method (Rotem at al., 2015 PNAS). We and others have used this assay in multiple papers so there is no need for “additional readouts”. Representative images, measurements of sphere size, and number spheres formed are irrelevant given the assay used in the paper.

3. Again, this is a standard growth assay, and of course the measurement is done before cells have reached confluence. Additional readouts for growth are inferior and irrelevant.

4. Unclear what “statistics” are requested, especially as we believe that we have provided such analysis.

5. Knock-outs and siRNA knockdowns are standard genetic methods that address biological functions, but they have pros and cons for specific experiments. We prefer siRNA knockdowns for transcriptional experiments because the short time frame makes them better for determining direct effects. Knockouts take a longer time for establishing cell lines, and this winds up with more indirect effects.

6. Co-IP, especially on recombinant proteins, is certainly sufficient to prove that the proteins interact and the similar genome association profiles are consistent with this interaction. Immunostaining is an inferior method given the low resolution, and proximity-ligation assays are much more complicated and not needed.

7. This comment is incorrect because ß-catenin levels are not decreased in the +TAM condition; they are the same. However, it is true that we see increased ChIP signal of ß-catenin in the +TAM condition. We don’t really know why, but an obvious possibility is post-translational modification (e.g., phosphorylation) in +TAM that increases specific binding. This is now mentioned in the revised paper.

8. The expression profiles in the ß-catenin, WDR77, MafB, and TCF4 depletion experiments are similar to each other and to expression profiles in depletions of other proteins important for transformation in our model. This is because anything that affects the positive feedback loop involved in transformation gives very similar gene expression profiles. This has been extensively discussed in our previous papers (cited in the present paper), and indeed GSEA analysis has been done before. qPCR analysis on a few selected genes is unnecessary and basically useless. The basic results are clear.

9. I don't understand this comment. The knockdowns do affect some ß-catenin target genes, but this is true for all such knockdowns that affect transformation. I don’t understand the relevance of the TOP/FLASH assays to this.

10. Why should we provide more than 1 example? The only point of a representative example is to show what the data looks like in a simple way. The heat maps and the analyses are based on all examples, which is much more informative.

11. While our Src-inducible model is clearly artificial, it has many technical advantages. More importantly, results over the last 13 years indicate their relevance to other cell line models and to human patient data. As stated above, this includes 33 publication that have been cited around 6000 times.

Minor comments heeded where appropriate.

**Reviewer 2**

0. The summary paragraph misunderstands the conclusions of the paper. First, we did not claim that interactions between the 3 proteins were correlated with the level of paused Pol II and transcriptional activity. Instead, we claimed that the genomic binding sites of these proteins are similar to each other and to paused Pol II (and transcriptional activity, which is well known to be correlated to the level of paused Pol II; e.g. our recent eLife paper by Petrenko and Struhl). Second, we never claimed that these interactions (as opposed to the proteins) were relevant for transformation; quite the opposite, as we question whether they are. Third, we never claimed that there is a ternary complex; I have no idea where this came from. Fourth, we never claimed that “genomic occupancy, regardless of distance or proximity, reflect a paused Pol II to coordinate a response between these factors”; again, I don’t know where this came from. Fifth, we never claimed that the results support any aspect of underlying biology. Essentially all the criticisms of Reviewer 2 concern “conclusions” that we did not make. The real conclusions of the paper are the observations *per se*, and these are not contested

1. There is a major misunderstanding. We did not use the ER-Src model because of our recent YAP/TAZ and S100A8/A9 papers. Instead, we clearly indicated that we have used this model for numerous papers, and the model has been well validated over the past 13 years. The only relevance of the YAP/TAZ and S100A8/A9 papers is that they both involve transcriptional co-activators. Other than this, there are no sentences whatsoever linking the results of the present paper to YAP/TAZ.

2. While some co-IP results in extracts are more robust than others, the results are clear. More importantly, direct interactions are validated using recombinant proteins.

3. I don’t understand the criticism of the ChIP-seq experiments. The main results are obvious upon inspection, and indeed are not criticized. While it is known that ß-catenin ChIP signals are relatively weak, this is irrelevant. Absolute crosslinking efficiencies are affected by many parameters; what matters is where the peaks are. Surely, Reviewer 2 does not think that the striking similarity of the ß-catenin, MafB, and WDR77 target sites is a random coincidence. In addition, Reviewer 2 did not appreciate our multiple lines of evidence against a technical “artifact”, namely the antibody and protein specificity and most importantly the striking specificity of target site location (i.e., near paused Pol II). We never argued that the two-step method was inherently better for avoiding artifacts; on the contrary we worried about this. Lastly, while we think that a technical artifact is highly unlikely (and no model for such an artifact is even mentioned), this does not mean that the results have biological significance, and we never claim this.

4. It is obvious upon inspection of Fig. 4 that most ß-catenin, MafB, and WDR77 target sites map very close to paused Pol II. Paused Pol II is defined simply by its location (strong peak) just downstream of transcription start sites. This definition has been used by numerous investigators over the past 15 years. Importantly, we do not claim any other relationship between ß-catenin, MafB, and WDR77 to paused Pol II beyond the close location of the target sites. We do discuss possible explanations for why these 3 proteins associate very near paused Pol II, but we do not make any claims

5. Related to point 4, the correlation between ß-catenin/MafB/WDR77 and paused Pol II binding levels is a simple fact based solely on the actual binding data. It is well known that levels of paused Pol II are well correlated with transcription levels (e.g., Petrenko and Struhl eLife 2021). Thus, it directly follows that genes bound by ß-catenin/MafB/WDR77 have higher expression levels than genes not bound by these proteins. While these results are clear, they do not indicate, and we do not claim, that these binding events are functionally significant. Indeed, ß-catenin/MafB/WDR77 target sites are not clearly linked to differential expression upon depletion, so the functional significance is unclear. We were extremely careful to discuss this in the paper. To put it differently, Reviewer 2 is criticizing us for not providing evidence for claims we did not make.

**Reviewer 3**

We really appreciate this Review, particularly for understanding the strengths and weakness of the paper. This reviewer does not contest any of the observations and indicated that no new experiments are necessary or suggested. The revised manuscript fixes the issues mentioned, which are merely clarifying and do not affect the results or conclusions.

Sincerely yours,

Kevin Struhl
