# Supplementary material for: MafB, WDR77, and ß-catenin interact with each other and have similar genome association profiles
Source: PLoS One. 2022 Apr 28;17(4):e0264799. doi: 10.1371/journal.pone.0264799 (PMC9049301; doi:10.1371/journal.pone.0264799)
Supplement: Attachment — Submitted filename: renamed_fd269.doc [file pone.0264799.s003.doc]

April 4, 2022

Dear Editors,

Attached is a revised version of our paper still entitled “**MafB, WDR77, and ß-catenin interact with each other and have similar genome association profiles**” (RC2021-01184) for publication in PLOS ONE. I should clarify that the original manuscript was sent to Review Commons and was then transferred to PLOS ONE. The original response was to the reviewers of the manuscript submitted to Review Commons. Here, the response is to the single reviewer of the transferred PLOS ONE manuscript, who suggested a few very minor changes, which we have heeded. Point-by-point responses below.

**Reviewer 1**

1. We agree that readers should not have to go to our previous papers to understand technical aspects of our model. As requested, we added several sentences at the beginning of the results section (first paragraph) that described the key features of the model. The mass spectrometry experiments are now described in the methods, and a table of interacting proteins is now presented (this was an unintended omission for the previous version).

2. Most of the binding sites are those described in detail in the paper. We don’t think it is particularly useful to discuss the other sites as we haven’t learned much. As for the
GO/KEGG analysis of regulated genes, we pointed out in our previous response that the expression profiles in the ß-catenin, WDR77, MafB, and TCF4 depletion experiments are similar to each other and to expression profiles in depletions of other proteins important for transformation in our model. This is because anything that affects the positive feedback loop involved in transformation gives very similar gene expression profiles. This has been extensively discussed in our previous papers (cited in the present paper), and indeed GO/KEGG analysis has been done before. In terms of transcriptional and functional pathways, the results in this paper do not add anything beyond the fact that ß-catenin, WDR77, MafB, and TCF4 are part of the transformation pathway.

Minor points: All as requested. We added details about tamoxifen in both the results and methods section. I don’t know what statistical analysis is desired in Fig. 1D,E; we show error bars and ±SD of 3 replicates. We deleted H3K27ac from the description of Fig. 4C. We fixed the typographical errors in the Figures (I’m impressed the Reviewer noticed them; we didn’t).

Sincerely yours,

Kevin Struhl
